# Exposure to artificial light at night mediates the locomotion activity and oviposition capacity of *Dastarcus helophoroides* (Fairmaire)

**DOI:** 10.3389/fphys.2023.1063601

**Published:** 2023-02-09

**Authors:** Xiang-lan Jiang, Zhe Ren, Xiao-xia Hai, Ling Zhang, Zhi-gang Wang, Fei Lyu

**Affiliations:** ^1^ Key Laboratories for Germplasm Resources of Forest Trees and Forest Protection of Hebei Province, College of Forestry, Agricultural University of Hebei, Baoding, Hebei, China; ^2^ Chengde Academy of Agriculture and Forestry Sciences, Chengde, Hebei, China

**Keywords:** Dastarcus helophoroides, locomotor activity, circadian rhythms, oviposition, light pollution

## Abstract

Light entrains the endogenous circadian clocks of organisms to synchronize their behavioral and physiological rhythms with the natural photoperiod. The presence of artificial light at night disrupts these photoperiodic cues and is currently considered to be a major threat to key fitness-related behaviors, including sleep disruption and physiological stress. Research on the ecological influence of forest pest and their natural enemies is lacking. The wood-boring insects significantly damage forest and urban forest ecosystem functions. The parasitic beetles, *Dastarcus helophoroides* is an important natural enemy of wood-boring insects, especially those in the Cerambycidae family. However, the effect of artificial light at night on the locomotor rhythms and oviposition capacity of *D. helophoroides* has received little attention. To address this gap, diel changes in the locomotor activity and number of eggs laid by female *D. helophoroides* was analyzed under different light-dark (LD) cycles and temperatures. The results showed that the 24-h rhythmic of locomotor activity in these beetles was elevated in darkness and reduced under illumination, indicating that they are nocturnal insects. This activity has two major peaks, the evening (1–8 h after lights off) and morning (3.5–12.5 h after lights off) components, reflecting that light mediate regular changes in locomotor activity. Moreover, the circadian rhythms and active percentage were influenced by the illumination duration and temperature, especially constant light and 40°C. Females laid more eggs under the 16 L: 8 D cycles at 30°C than under the other combinations of photoperiod (including constant light and darkness) and temperature. Finally, the potential influence of exposure to four ecologically relevant intensities of artificial light at night (0, 1, 10 or 100 lx) on oviposition capacity was studied*.* The results showed that lifetime exposure to bright artificial light (1–100 lx) at night decreased the number of eggs laid relative to those laid with no lighting at night. These results demonstrate that chronic exposure to bright artificial light at night may influence the locomotor activity and oviposition capacity of this parasitic beetle.

## Introduction

Before the invention of electric lighting, mammals and invertebrates spent nearly all of their time during the day exposed to natural daylight (>300 lx) and nearly all of their time after sunset exposed to dim sources of light (<30 lx), including moonlight, starlight and small fires (Phillips et al., 2019). Earth’s rotation produces daily cycles of light and dark, influencing the behaviors, physiological functions, metabolic regulation, and hormone secretion of organisms, and even plant rhizosphere microbial communities (Zhao et al., 2021; Liu et al., 2022; Mason et al., 2022; Sanders et al., 2022). As a result, these behaviors and physiological processes show an inherent ability to follow the change in light/dark cycles (LD cycles) at approximately a circa 24-h scale. Therefore, an organism’s behavioral and physiological processes, such as locomotor activity, food intake, and sleep, display rhythmic oscillations in response to the internal circadian clocks (Brüning et al., 2015; Mason et al., 2022). Circadian clock is significantly influenced by the alteration of natural light environment, for example, the daily LD cycle (Brüning et al., 2015).

With rapid urbanization, natural light regimes have been widely disrupted by artificial light at night, such as streetlights, industrial and domestic lighting and vehicle lighting (Sanders et al., 2022). Artificial light has transformed the nighttime environment of large areas of the Earth; 23% of the world’s land surfaces, 88% of Europe, and almost half of the United States are estimated to be exposed to light pollution, which includes light sources such as small night lights indoors, television light indoors, streetlights, industrial lighting and vehicle lighting (Falchi et al., 2016). Exposure to light at night has been reported to influence the circadian clock and health of humans and animals. Compared to the absence of light exposure during sleep, exposure to any artificial light in the bedroom during sleep was associated with obesity (in women and older age groups), cardiometabolic syndrome, type 2 diabetes (in an elderly population), and depression (in young people) (Park et al., 2019; Obayashi et al., 2020; Crouse et al., 2021; Kim et al., 2022; Mason et al., 2022). In birds, mammals, and fruit flies, individual fitness traits, such as reproduction and juvenile growth, were reduced by chronic exposure to light at an intensity less than 10 lx (Kempenaers et al., 2010; McLay et al., 2017).

Comparatively, few studies have investigated whether fitness in invertebrates is impacted by exposure to dim nighttime lighting (McLay et al., 2017). Previous studies have shown a negative effect of constant light exposure on fecundity and longevity in a model species (*Drosophila melanogaster*) relative to a normal day-night environment (Kouser et al., 2014). Female *D. melanogaster* chronically exposed to light at night (light intensities of 1, 10 and 100 lx) were less likely to commence oviposition than females exposed to 0 lx light at night (McLay et al., 2017). However, knowledge of how the reproductive capacity of non-model invertebrates is influenced by light exposure at night remains limited.

The beetle *Dastarcus helophoroides* (Fairmaire) is an important generalist or polyphagous parasitoid of several longhorn beetle species in China, Korea, and Japan, including *Anoplophora glabripennis* Motschulsky, *Monochamus alternatus* (Hope), *Massicus raddei* Blessig and *Batocera horsfieldi* (Hope), and has been widely used to control Cerambycidae beetles (Wei et al., 2013; Lyu et al., 2014; Yang et al., 2014; Kaitlin et al., 2018; Shen et al., 2022). Under natural LD cycles at a temperature of 27°C ± 1°C, the locomotor activity of *D. helophoroides* showed an obvious circadian rhythm, peaking within 0.5–2.5 h after lights off during the dark period (Lyu et al., 2015a). A similar rhythm of locomotor activity was revealed under a LD cycles of 16:8 h at 23°C ± 1°C in an artificial climate chamber (Lyu et al., 2015b), but the locomotor active percentage of adults at 27°C ± 1°C was approximately equal to 1.5 to 2 times that at 23°C ± 1°C at peak time. However, the influence of exposure to different illumination durations at night and temperatures on the locomotor activity of this beetle is still unknown.

The beetle *D. helophoroides* is an ideal model species for examining the influences of artificial light on behavioral and physiological traits. Because it is a nocturnal insect and its prey (Cerambycidae beetles) is widely distributed in different urban and rural habitats (Lyu et al., 2014), these beetles are affected by different intensities of artificial night lighting in urban and rural environments. Moreover, recent studies have revealed decreased parasitic efficiency of *D. helophoroides* in controlling *M. alternatus* in southern pine forests, especially in forests containing dying *Pinus massoniana* (Lamb.) caused by the longhorn beetle (Shen et al., 2022). The number of eggs laid by insects is often negatively affected by several ecological factors, such as exposure to light at night and extreme temperatures (Yang et al., 2012; McLay et al., 2017). Therefore, a series of experiments were performed to investigate the effects of different illumination durations at night and different temperatures on the two key life history traits (locomotor activity and oviposition capacity) in a non-model invertebrate, *D. helophoroides*. In addition, the potential effect on the number of eggs was determined when adults were exposed to four different ecologically relevant intensities of artificial light at night.

## Materials and methods

### Insects

Adults of the beetle *D. helophoroides* were sourced from the Department of Entomology, Agriculture Vocational College of Beijing. The first generation of the wild population was collected from parasitized larvae and pupae of *A. glabripennis*. Larvae were reared on a substitute host (*Thyestilla gebleri* (Fald.). Adults used in the experiment were the second generation of beetles and maintained in white plastic cages in the laboratory at 25°C ± 1°C and 50% ± 10% relative humidity under an LD cycle of 16:8 h (light: 500 lx and dark 0 lx) in an artificial climate chamber (RXZ-500D, Jiangnan Instrument Factory, Ningbo, China). Plastic centrifuge tubes (diameter: 10 mm and length: 50 mm) were plugged with a cotton ball to provide water, and *Tenebrio molitor* (L.) larvae oven-dried at 60°C served as food for the adults. The water and food were changed every 5 days. Female adults were used in behavioral tests and distinguished by the end angle of the anal plate and the length and width of the anal plate under a dissecting microscope (Olympus SZ51, Tokyo, Japan) according to Tang et al. (2007). All adults that did not differ in body size were used in our experiment at 60–70 days after emergence, and all of the individuals were able to mate and oviposit normally in our experiment.

### Locomotor activity recording

**Figure 1:**
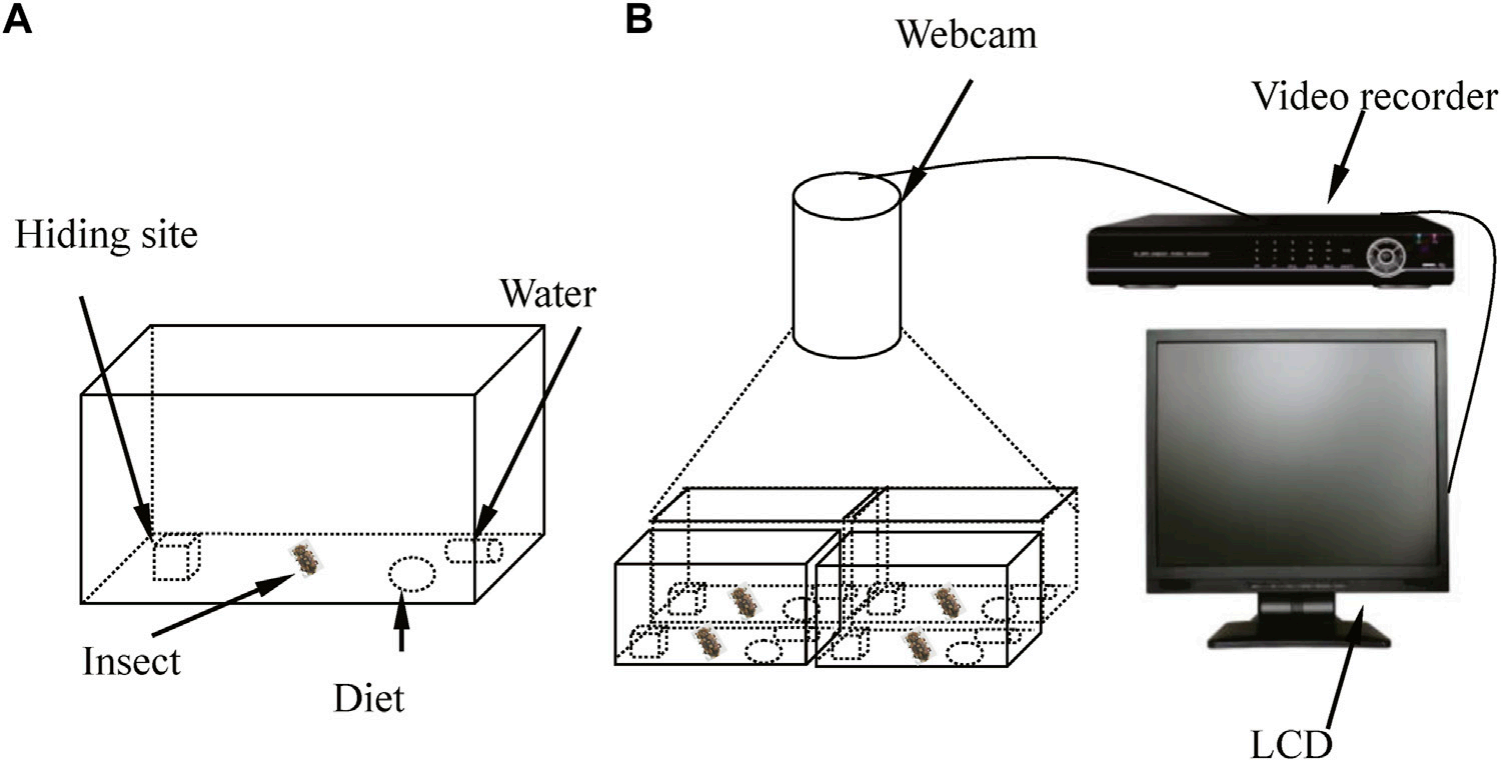
Schematic of the behavioral bioassay chamber used to monitor the circadian rhythms of locomotor activity by female adults under different conditions. **(A)** Females *D. helophoroides* were placed in the breeding box during the experiment. **(B)** The equipment used to observe the locomotor activity of female adults during the experiment. LCD: liquid crystal display.

Based on the behavioral characteristics of *D. helophoroides* adults in a previous experiment (Lyu et al., 2015a), equipment was designed to observe and record the circadian rhythm of locomotor activity under different temperatures and LD cycles (Figure 1). The experimental equipment comprised four artificial breeding boxes, a video camera (high definition 720P), a liquid crystal display, a hard disk video recorder and a coaxial cable (Figure 1). The artificial breeding box was designed as shown in Figure 1A. The breeding box consisted of a polyvinyl box (18.5 cm × 12.5 cm × 7.5 cm), wooden block with a concave trough (3 cm × 3 cm × 3 cm), artificial diet and water resource. In a previous study (Lyu et al., 2015a), 55%–100% of adults hid under the wooden block during the light period; thus, a wooden block with a concave trough was placed in a polyvinyl box to provide a resting site. To prevent the test insects from escaping and to observe their activity, a layer of odorless plastic wrap with 50–100 pinholes was placed on the top of a breeding box to allow air circulation. The video camera in the artificial climate chamber, hard disk video recorder and liquid crystal display were connected by the coaxial cable to record locomotor activity.

### Locomotor activity

#### Effect of LD cycle on the locomotor activity

The beetle *D. helophoroides* is a nocturnal insect. These beetles show characteristic clock-controlled evening locomotor activity at temperatures of 23°C ± 1°C and 27°C ± 1°C (Lyu et al., 2015a; Lyu et al., 2015b). However, whether the rhythm of locomotor activity is influenced by the duration of illumination at night remains unclear. The previous study showed no significant difference in locomotor activity between females and males under different conditions (Lyu et al., 2015a; Lyu et al., 2015b). In addition, the effect of different microhabitat conditions on the oviposition capacity of beetles was measured in the second section. Therefore, the locomotor activity of female beetles, which is more correlated with oviposition behavior (relative to that of male beetles), was monitored at a temperature of 25°C under seven different LD cycles to examine how illumination duration at night affects the rhythm of locomotor activity (in hours, from constant darkness to constant light), including constant darkness (0 L/24 D), 4 L/20 D, 8 L/16 D, 12 L/12 D, 16 L/8 D, 20 L/4 D, and constant light (24 L/0 D), all measured in hours. Locomotor activity mainly includes the moving, foraging and drinking behavior of beetles. The recorded data was observed every 30 min, and locomotor activity at a time point was confirmed if the beetle was found at a different location from the previous location. Individual female beetles were placed in an artificial breeding box under the different LD cycles, and locomotor activity under these different conditions was recorded for continuous 10 days. Twenty individual beetles were tested per LD cycles.

#### Effect of temperature on the locomotor activity

Previous studies also showed that at the peak time of locomotor activity, the percentage of adult beetles displaying this behavior at 27°C ± 1°C was approximately 75% of the total beetles; however, at a temperature of 23°C ± 1°C, this percentage decreased considerably to 35% (Lyu et al., 2015a; Lyu et al., 2015b). To determine how different temperatures affect the rhythm of locomotor activity, the locomotor activity of females was monitored under the LD cycle of 16:8 at six temperature treatments, including 15°C, 20°C, 25°C, 30°C, 35°C, and 40°C. Individual female beetles were placed in an artificial breeding box under these different conditions, and then their locomotor activity was recorded for continuous 10 days. Twenty individual beetles were tested per temperature condition.

### Oviposition capability

#### Effect of the LD cycle and temperature on the number of eggs laid

A series of experiments were conducted to establish the effect of exposure to different illumination duration at night and different temperatures on the number of eggs laid by female beetles in closed arenas (i.e., glass Petri dishes), including the above mentioned seven different LD cycles and six different temperatures (10°C, 15°C, 20°C, 25°C, 30°C, 35°C). Glass Petri dishes (14 cm diameter, 2.5 cm height) were used in the experiment. We pasted white filter paper to the wall and bottom of each glass Petri dish to visually isolate individuals, and thereby prevent the different groups from influencing each other. Wooden blocks (1.5 cm high × 1.5 cm wide × 1.5 cm long), each with a carved trough, were placed on top of the filter paper to provide a microhabitat for oviposition. The glass Petri dishes were then placed in an artificial climate chamber under the previously described microhabitat conditions. Three female and three male beetles were introduced in the center of the experimental arena (the glass Petri dishes) to determine female oviposition. The adults remained in glass Petri dishes for a period of 30 successive days to allow oviposition to occur. Female *D. helophoroides* usually laid clusters of eggs on the filter paper beneath the wood blocks. Every 24 h, the number of eggs on the filter paper was counted under a dissecting microscope (Olympus SZ51, Tokyo, Japan). Then, the frass of adults and food debris were cleared from the glass Petri dishes to decrease their influence on the next count. Fifteen groups (15 replicates × 3 male-female adult pairs) were tested per condition.

#### Effect of light intensity at night on the number of eggs laid

To determine the influence of light at night on oviposition, four light conditions were created in an artificial climate chamber, each with identical daytime lighting conditions (500 lx) but varying in the intensity of nighttime lighting as follows: 0 lx (control, complete darkness at night), 1 lx (10 times full moonlight on a clear night), 10 lx (similar to nighttime street illumination), and 100 lx (similar to bright urban lighting) (Brüning et al., 2015; Botha et al., 2017). The results of the experiment described in section *“Effect of LD cycle and temperature on the number of eggs laid”* indicated that beetles laid more eggs under an LD cycle of 16:8 at 30°C than under the other treatments (Figure 5); therefore, an LD cycle of 16:8 and a temperature of 30°C were selected to evaluate the effect of the intensity of light at night on the number of eggs laid. The number of eggs laid on the filter paper was counted each day. Fifteen groups (15 replicates × 3 pairs of adults) were tested per treatment.

### Statistical analyses

In each set of experiments, to assess the influence of the LD cycle and temperature on locomotor activity, the means of the activity percentage during the light or dark phases were compared using one-way analyses of variance (ANOVAs) and Duncan’s *post hoc* tests. The mean activity percentages between the light and dark periods in the different treatments were compared by means of a two-tailed independent-samples Student’s t-test or Mann-Whitney test if the data displayed severe heteroscedasticity. Moreover, one-way ANOVAs were used to assess differences in the average number of eggs laid under the seven different LD cycles, six different temperatures and four different light intensities at night. Duncan’s *post hoc* test was used to determine significant differences between groups. All statistical analyses were performed using IBM SPSS Statistics 21.00 for Windows (IBM SPSS Inc., Boston, MA, United States).

## Results

### Locomotor activity

#### Effect of LD cycle on the locomotor activity

**Figure 2:**
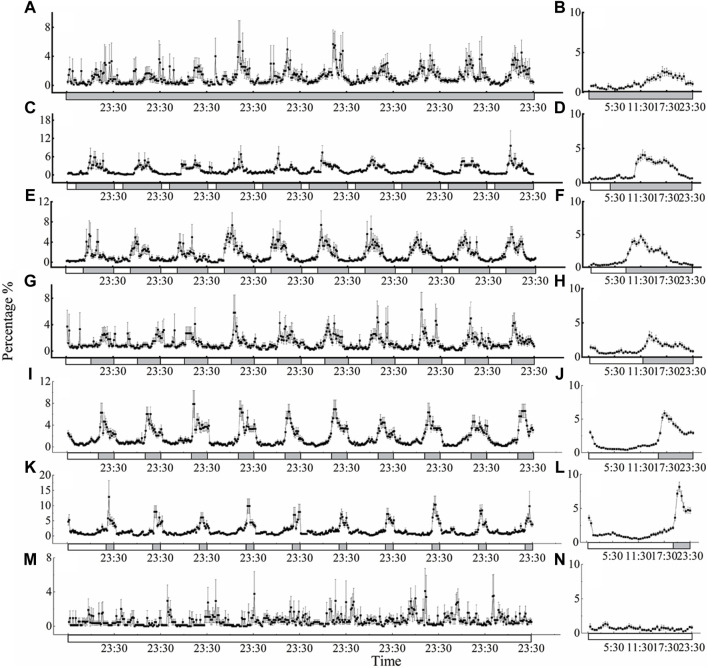
Circadian rhythms of locomotor activity of *D. helophoroides* under different LD cycles at a temperature of 25°C. LD cycles: **(A, B)** 0L/24D; **(C, D)** 4L/20D; **(E, F)** 8L/16D; **(G, H)** 12L/12D; **(I, J)** 16L/8D; **(K, L)** 20L/4D; **(M, N)** 24L/0D. Data (n = 20) shown are the mean ± standard error of the mean (SEM). The percentages in the figure indicate the occurrence frequency of single behaviors of individual insects at 30 min various time intervals within 1 day. Black and white bars represent the LD cycle, black = subject night, white = subject day.

The locomotor activity of *D. helophoroides* was monitored under seven different LD cycles at a temperature of 25°C (Figure 2). Under the different LD cycles at 25°C, there were two major peaks in locomotor behavior in the morning and evening, except for the constant light condition (Figure 2). The highest peak in locomotor activity occurred in the dark period from 2 h to 16.5 h after lights off signal, and the second peak in locomotor activity occurred at ZT = 16:00–23:30 before “lights on signal” (ZT0 = lights on; Figure 2 B, D, F, H, J, and L). There was a direct correlation between the locomotor activity peak after lights off signal and the duration of the light period, with the peak activity moving progressively later with increasing durations of the light phase (Figure 2 D, F, H, J, and L). The locomotor activity peak of female occurred 16.5 h after lights off signal under the constant darkness condition. When the duration of illumination was increased, the timing of the first peaking in the locomotor activity were shifted forward relative to that under the constant darkness condition; for example, under the LD 4:20 cycle, the timing of the first peak in locomotor activity was 8.5 h after lights off signal; whereas the timing of the first peak under the LD cycles of 8:16, 12:12, 20:4 was 2.5 h, 2 h, and 2 h after lights off signal, respectively (Figures 2B, D, F, H, J, L). In contrast, the beetles showed no circadian regulation of locomotor activity under the constant light condition (Figures 2M, N).

To further probe the influence of different LD cycles, the locomotor active percentages was analyzed in the light and dark phases under the different conditions (Figure 4 A). The average active percentages of adults in the dark phase were significantly higher than that in the light phase (Supplementary Table S1 4.089 < *t* < 21.411, *p* < 0.001), except under the constant light and darkness conditions. Furthermore, the active percentages of adults were significantly higher under the LD cycles of 16:8 and 20:4 than under the other LD cycles at a temperature of 25°C (Figure 4A). The active percentages of adults in the dark phase were 80.06% and 76.94% under the LD cycles of 16:8 and 20:4, respectively, and there was no significant difference in the active percentages in the light phase and dark phase between the LD cycles of 16:8 and 20:4 (Figure 4A; Supplementary Table S1).

#### Effect of temperature on the locomotor activity

**Figure 3:**
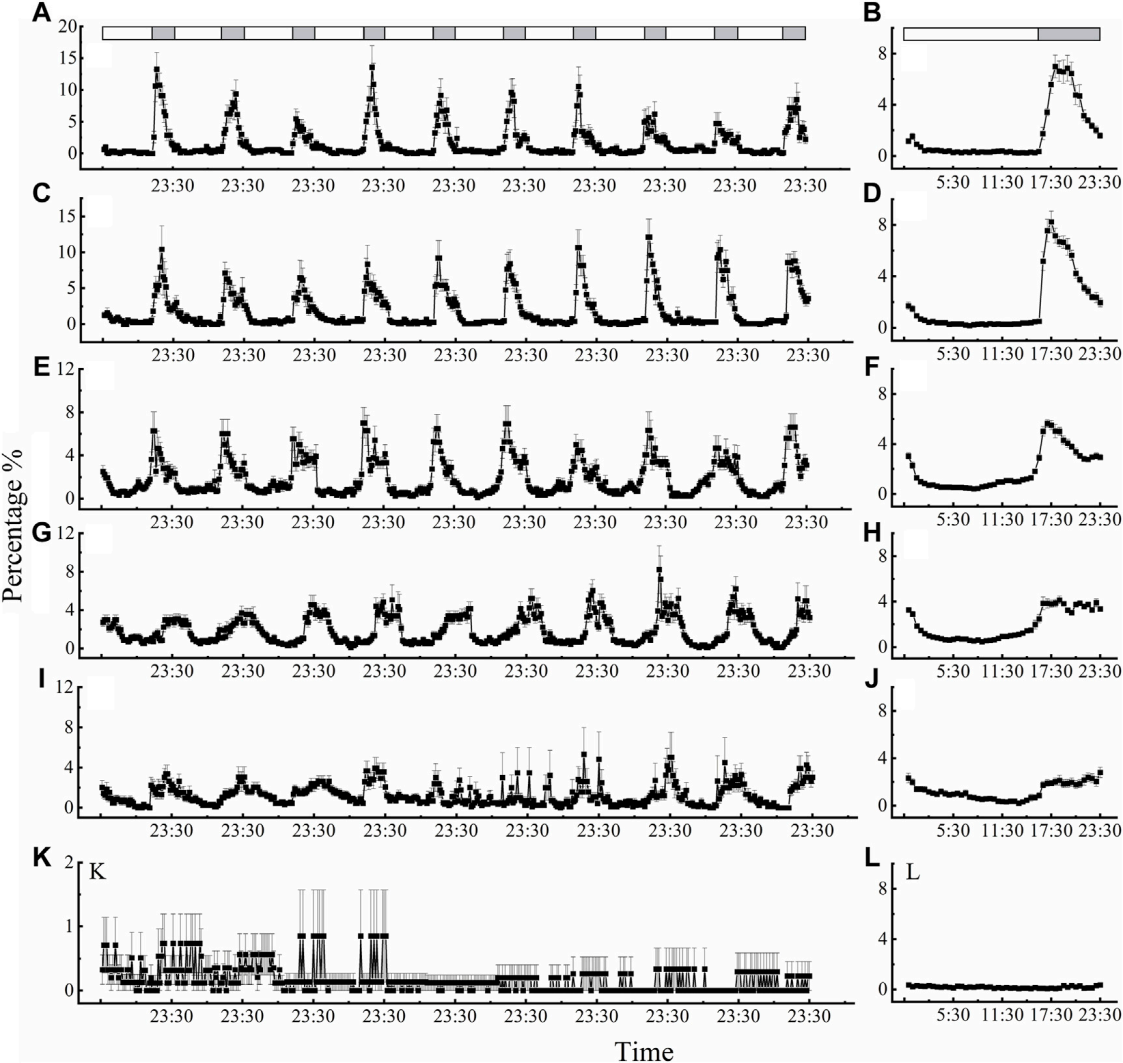
Circadian rhythms of locomotor activity of *D. helophoroides* at different temperatures. **(A, B)** 15°C; **(C, D)** 20°C; **(E, F)** 25°C; **(G, H)** 30°C; **(I, J)** 35°C; **(K, L)** 40°C. Data (n = 20) shown are the mean ± SEM. The percentages indicate the occurrence frequency of single behaviors of individual test insects in various time intervals within 1 day. The light-dark cycle was 16 h L/8 h D. Black and white bars represent the LD cycle, black = subject night, white = subject day.

Locomotor activity also displayed regular rhythms under the LD 16:8 cycle at different temperatures, except for 40°C (Figure 3). The beetles showed characteristic clock-controlled evening locomotor activity, peaking between ZT 17:00 and ZT 18:30 (ZT0 = lights on; Figure 3 B, D, F, H and J). The first peak in locomotor activity occurred approximately 2.5 h, 2 h, 1.5 h, 3 h after lights off signal at 15°C, 20°C, 25°C and 30°C, respectively. Compared with performance of adults under dark phase at 15, 20, 25, 30, 35°C (Figures 3B, D, F, H, J), the rhythm of locomotor activity at 40°C was irregular, smooth, and exhibited only slight changes (Figure 3L). Moreover, we also found a subtle small peak in activity before “lights on” (ZT = 23:00–23:30 after lights on) at temperatures of 15°C, 20°C, 25°C, 30°C, and 35°C, which was labeled the “morning peak” (Figures 3A, C, E, G, I, K).

**Figure 4:**
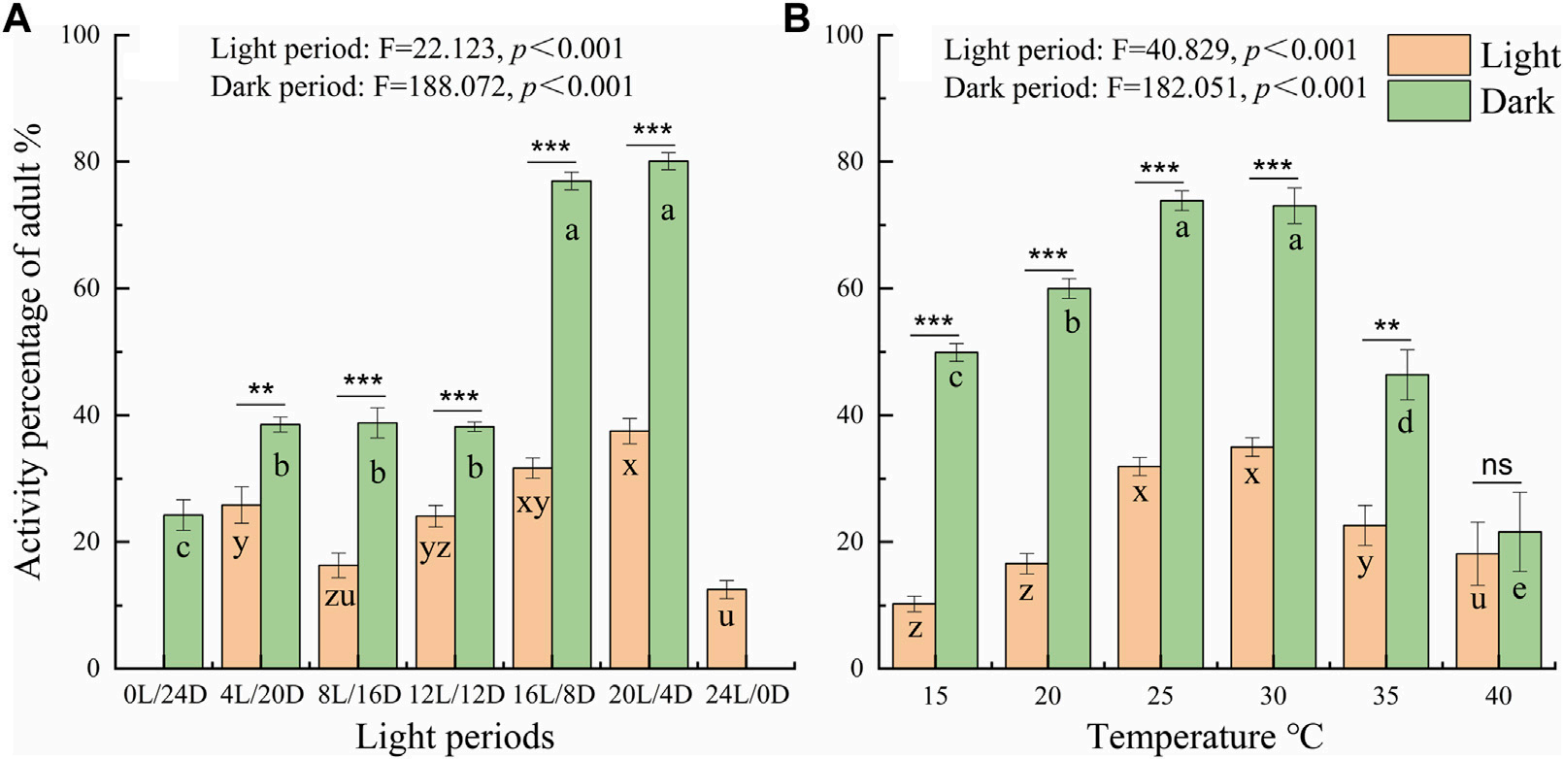
Comparison of the locomotor active percentages of *D. helophoroides* between the light and dark phases under different LD cycles **(A)** and temperatures **(B)**. The difference in active percentage between the light and dark phases under the different conditions was determined by two-tailed Student’s t tests (***p < 0.001, **p < 0.01). a, b, and c indicate significant differences in the active percentages of during the dark phase at different temperatures according to Duncan’s post hoc test, one-way analysis of variance (α = 0.05). x, y, and z indicate significant differences in the active percentage during the light phase at different temperatures according to Duncan’s post hoc test, one-way analysis of variance (α = 0.05). Data shown are the mean ± SEM (n = 10).

At the different temperatures under the LD cycle of 16:8, the active percentages in the dark phase were significantly higher at 25°C and 30°C than at the other temperatures (Figure 4B). The active percentages of females in the dark phase at temperature of 30°C and 25°C were 79.56% and 75.38%, respectively. There was no significant difference in the active percentages of females between 30°C and 25°C (Figure 4B; Supplementary Table S2). The average active percentages of adults in the dark phase were significantly higher than those in the light phase (Supplementary Table S2 3.507 < *t* < 26.809, *p* < 0.003), except at 40°C (Supplementary Table S2 *t* = 0.194, *p* = 0.848).

### Oviposition capability

#### Effect of LD cycle and temperature on oviposition capacity

**Figure 5:**
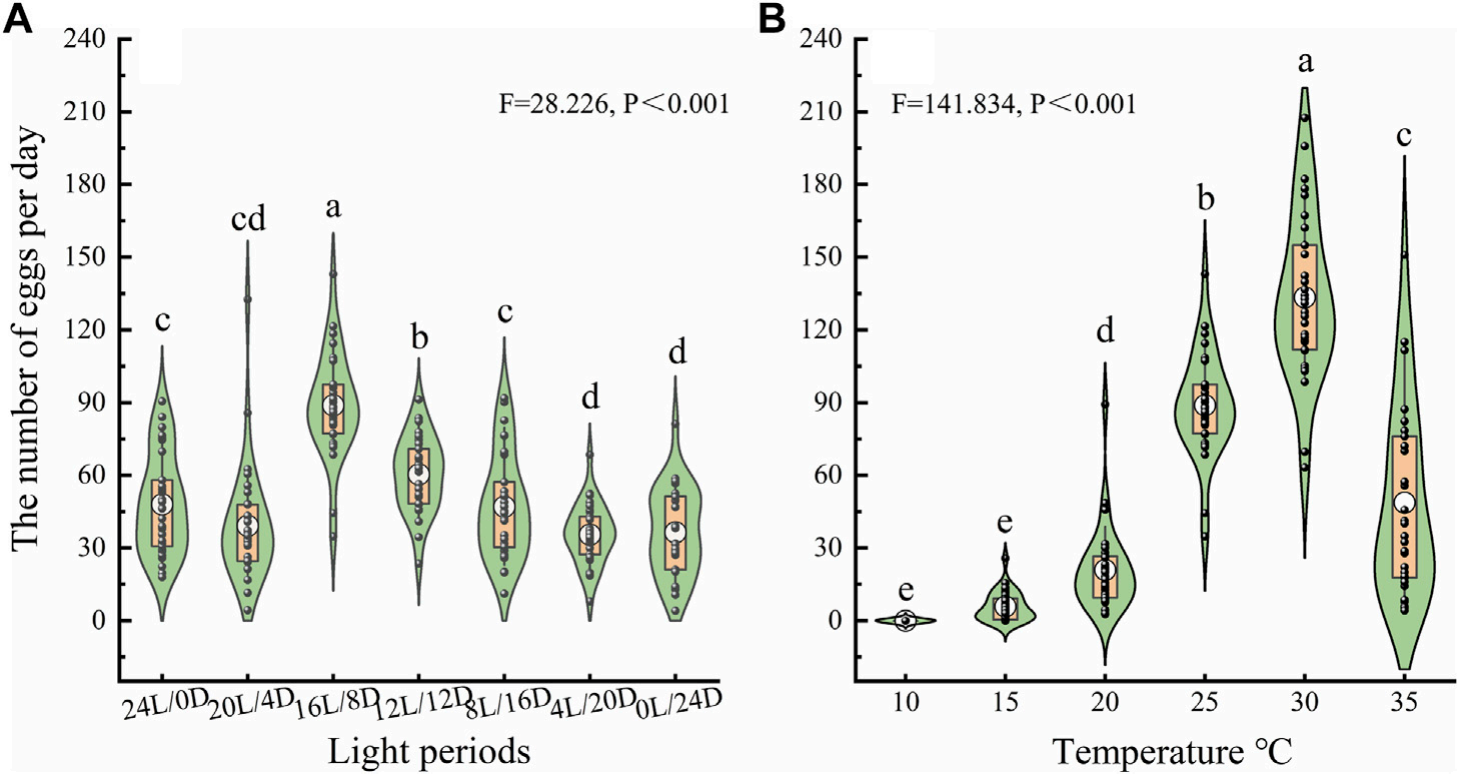
Number of eggs laid by ovipositing females under different LD cycles **(A)** and temperatures **(B)**. The lower and upper vertex of lines within the violin plots and the lines of the lower and upper edges of the box indicate the 10%, 90%, 25% and 75%, respectively; the middle circles in the boxes indicate the mean number of eggs laid. a, b, and c indicate a significant difference in the number of eggs laid among different conditions according to Duncan’s post hoc test and one-way analysis of variance (α = 0.05).

To investigate the oviposition capacity of female beetles, a series of bioassays were conducted under the different treatments, The number of eggs laid on the glass Petri dishes was affected by the LD cycle and temperature (Figure 5). The average numbers of eggs laid by females were 47.97 ± 3.76, 39.20 ± 4.43, 88.94 ± 3.93, 60.04 ± 2.79, 47.04 ± 3.95, 35.59 ± 2.26 and 36.44 ± 3.29 under LD cycles of 24:0, 20:4, 16:8, 12:12, 8:12, 4:20 and 0:24, respectively. Females laid far more eggs under LD cycle 16:8 than under the other cycles at 25°C (Figure 5A). The average numbers of eggs laid by females were 0, 5.89 ± 1.11, 20.93 ± 3.13, 88.94 ± 3.93, 133.38 ± 6.21, and 48.76 ± 7.16at 10°C, 15°C, 20°C, 25°C, 30°C and 35°C, respectively, under the LD cycle 16:8. Females laid significantly more eggs under LD cycle 16:8at 30°C than at the other temperatures (Figure 5B).

#### Light exposure at night decreases the number of eggs laid

**Figure 6:**
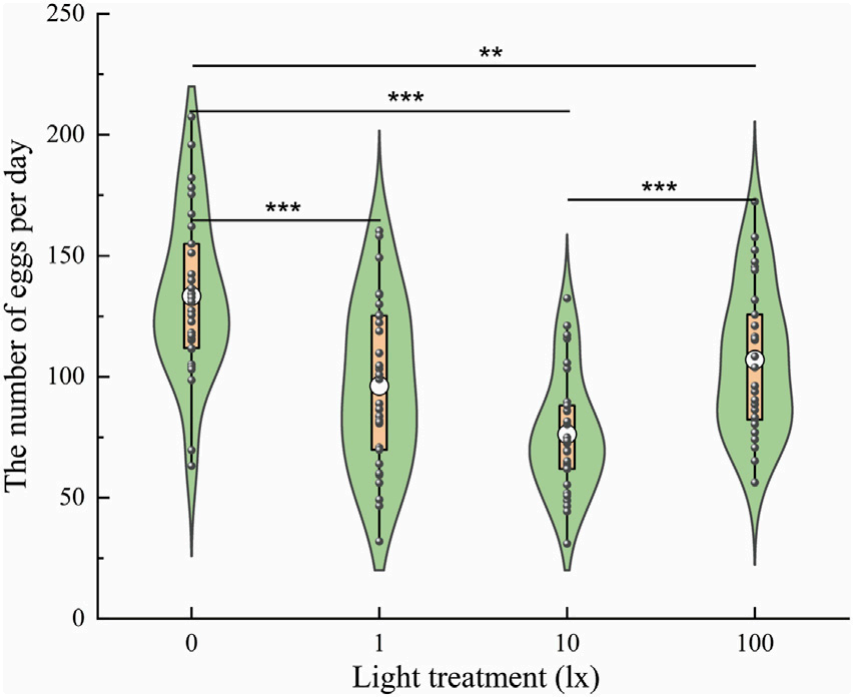
Number of eggs laid by ovipositing females the four light intensities at night. The lower and upper vertex of lines within the violin plots, the lines of lower and upper edges of boxes indicate 10%, 90%, 25% and 75%, respectively; the middle circles in the box indicate the mean number of eggs laid. An asterisk (*) indicates a significant difference in the number of eggs laid among the different conditions according to Duncan’s *post hoc* test and one-way analysis of variance (α = 0.05, ***p < 0.001, **p < 0.01).

To explore the effects of light intensity at night on oviposition, the number of eggs laid was evaluated under light intensities of 0 lx, 1 lx, 10 lx, and 100 lx (Figure 6). The results showed that the number of eggs laid was significantly affected by light intensity (F = 17.528, *df* = 3, *p* < 0.001); specifically, the average number of eggs laid per three pairs of adults was 133.38 ± 6.21, 96.23 ± 6.29, 76.27 ± 4.52, and 107.03 ± 5.54 under light intensities of 0 lx, 1 lx, 10 lx, and 100 lx, respectively. Females exposed to light at night (1, 10, and 100 lx) laid fewer eggs than those exposed to no light (0 lx) at night (Figure 6). There was no significant difference in the number of eggs laid between the 1 lx and 10 lx conditions or between the 1 lx and 100 lx conditions. Females exposed to light at night laid fewer eggs with increases in the light intensity. However, females exposed to100 lx light at night laid more eggs than those exposed to 10 lx light at night.

## Discussion

The experiments revealed a surprising and intriguing change in the circadian rhythms of locomotor activity and the oviposition capacity of *D. helophoroides* under different LD cycles and temperatures. First, the beetles displayed rhythmic oscillation of locomotor activity under different conditions, except under the constant light and 40°C. Locomotor activity elevated in darkness and reduced under illumination, indicating that this species is nocturnal insect. Across LD cycles, the active rhythm of females was 38.53%–80.06% at dark phase in the different LD cycles and 25.81%–37.50% in the light phase (Figure 4A; Supplementary Table S1). Similar active percentages were observed under the LD 16:8 cycle at different temperatures (Figure 4B; Supplementary Table S2). The active percentage was also influenced by the LD cycle and temperature (Figure 3). Second, the fecundity of the beetles (provided with sufficient nutrient supply) was highest under LD cycle 16:8at a temperature of 30°C rather than 25°C. Finally, and perhaps most importantly, chronic exposure to dim nighttime lighting significantly reduced the oviposition capacity of *D. helophoroides*. Taken together, these findings suggest a negative impact of dim nighttime illumination in terms of both duration and intensity on the locomotor activity and oviposition capacity of the beetle *D. helophoroides*.

The beetle *D. helophoroides* is a widely distributed in China (Lyu et al., 2014), and its circadian rhythms may be influenced by the complex natural cycle in environment. For example, in fruit flies, rhythmic locomotor activity shows a more complex oscillation under natural environments than under laboratory LD cycles (Vanin et al., 2012). Although various combinations of LD cycles and temperatures were used to determine locomotor activity rhythm in this study, it remains unclear how the complex natural cycles in the environment affect locomotor activity.

Moreover, previous studies have shown that the active percentage decreased significantly after three consecutive days under constant darkness but increased to normal levels after the adults were transferred to another laboratory and maintained under a natural LD cycle (Wei et al., 2008). The results of the present study corroborate those of previous research (Wei et al., 2008), which showed that approximately 24% of beetles per 30 min interval exhibited locomotor activity under the constant darkness condition and that the active percentage was significantly lower under the constant darkness than under the other LD cycles (Figure 4); in contrast 77%–80% of adults showed locomotor activity behavior in the dark phase under LD cycles of 16:8 and 20:4, suggesting that the illumination (lights on and off signals) mediates locomotor activity.

The experiments showed that the 24-h rhythmic of locomotor activity of *D. helophoroides* adults peaked twice, once in the evening (1–8 h after lights off, or 16,5 h under the constant darkness condition) and once in the morning (3.5–12.5 h after lights off), indicating a regular circadian rhythm. These results corroborate those of a previous study that reported two major locomotor activity peaks in the evening and forenoon (before 11:00) under a natural light-dark environment (Wei et al., 2008). However, the subtle smaller peak in locomotor activity before “lights on” was not observed at high temperature (35°C and 40°C) (Figures 3K, M), suggesting that temperature can also impact the locomotor activity rhythm. Notably, there was a very high mortality rate at 40°C after 3 days. Therefore, this temperature was excluded from the following oviposition test. Furthermore, recent research has shown that the parasitic efficiency of *D. helophoroides* on longhorn beetles is decreased in southern pine forests; this decrease may have been caused by extreme temperatures because high temperatures have frequently occurred in southern China (Shen et al., 2022). In present study, we found that the number of eggs laid by females at 35°C was significantly lower than that at 25°C and 30°C (Figure 5); thus, high temperatures may lead to a decrease in the oviposition capacity of beetles, thereby reducing parasitic efficiency.

Circadian rhythms driven by biological clocks are expected to enhance organism fitness by adjusting their locomotor activity and metabolism to adapt to the external environment (Zhao et al., 2021). Importantly, the endogenous circadian clock depends on light to synchronize behavior and physiological activity of organisms with the external daily environment (Bumgarner and Nelson, 2021). It is therefore reasonable to infer that synchronicity of these rhythms would be less advantageous to organisms living under conditions of constant light or darkness (Zhao et al., 2021). In the present study, beetles maintained under constant light or darkness appeared to lose the rhythmicity of locomotor activity. Although locomotor activity showed a certain rhythmicity, the active percentage of beetles was significantly lower under constant light or constant darkness conditions than under the other LD cycles, suggesting that constant darkness or light can derange temporal adaptation and decrease organism fitness (Figure 4). Indeed, in the oviposition experiment, females laid fewer eggs under constant light or constant darkness conditions than under other LD cycles (Figure 5).

Chronic exposure to dim artificial light at night impacted locomotor activity and also decreased the oviposition capacity of the *D. helophoroides* (Figure 6). On average, the presence of dim lighting at night resulted in a 20%–43% reduction in the number of eggs laid per three pairs of beetles (Figure 6: three pairs of beetles laid an average of 134 eggs per day under the 0 lx condition at night compared to an average of 94 eggs laid by females exposed to light at night). Dim lighting at night represents a suboptimal mating environment, as variation in oviposition with light conditions has been observed in moths, fruit flies and fireflies (Van Geffen et al., 2015; Firebaugh and Haynes, 2016; McLay et al., 2017). A previous study found that mating behavior occurred at 19:00–22:00 (Wei et al., 2008); therefore, the presence of night lighting is likely to have a direct influence on the rhythm of mating, in turn, affecting oviposition capacity.

Multiple physiological mechanisms have been proposed to explain the relationships of nighttime light exposure with altered locomotor activity and fecundity *via* changes in sleep. Robust evidence from experimental studies indicates that nighttime light exposure has negative impacts on the activity of enzymes in the juvenile hormone (JH) biosynthetic pathway and on the circadian secretion of melatonin and biogenic amines (Zera and Cisper, 2001; Guy et al., 2013; Zera, 2016). The circadian rhythms of reproductive functions, such as courtship, mating, and gamete production, were regulated by oscillations in JH titers. In cockroaches and bark beetles, oscillations in JH titers mediated the production or release of sex pheromones to influence female or male calling signals (Kang et al., 2019; Fang et al., 2021; Chen et al., 2022). Moreover, melatonin has been identified in many insect tissues, including the head, brain, and compound eyes (Guy et al., 2013). In mammals, the circadian secretion of melatonin is important for the timing of circadian and seasonal rhythms, influencing sleep quality (Mason et al., 2022). Because melatonin levels exhibit a circadian rhythms with peaks during the dark phase, nighttime light exposure can suppress melatonin secretion, and several studies have suggested a link between the suppression of nighttime melatonin and the incidence of type 2 diabetes (Kim et al., 2022) and impaired cardiometabolic function (Mason et al., 2022). In silkworms, neuroanatomical evidence suggested that melatonin may influence JH signaling and is likely to regulate reproduction (Guy et al., 2013). Moreover, lighting at night suppresses melatonin secretion and may contribute to higher levels of oxidative stress, influencing gamete production. The potential physiological mechanism underlying these associations needs to be determined by future studies.

Furthermore, this experiment may indicate optimal lighting and temperature conditions for mass rearing of *D. helophoroides*, including those that postponing or increasing oviposition. The *D. helophoroides* is the most important natural parasitoid of wood borers, including longhorn and buprestid beetles, and mass rearing is quite challenging (Yang et al., 2014). Emerging evidence has indicated that temperature influences the fecundity of this beetle, which reaches a maximum under sufficient nutrient supply at 22–25°C (Yang et al., 2012). However, the present study shows that females laid more eggs under LD cycle 16:8at 30°C than under the other conditions. In previous research, the conditions contained of four different temperatures of 16°C, 19°C, 22°C, and 25°C (Yang et al., 2012); in contrast, the present study included six different temperature conditions (10°C, 15°C, 20°C, 25°C, 30°C and 35°C). Different gradients and ranges utilized in various studies may lead to discrepant results. Previous studies have reported that constant darkness disturbs the normal behavioral rhythms of beetles and suggested that appropriate illumination should be considered for mass rearing (Wei et al., 2008). Indeed, in the present study, females laid fewer eggs under constant light or constant darkness conditions than under the other LD cycle treatments, suggesting that LD cycles are key to mass rearing. Moreover, the results showed that females laid an average of 0 and six eggs at 10°C and 15°C, respectively; and thus, these temperatures may be used to prolong the shelf life of this beetle. Overall, the present study established a foundation for the mass rearing of *D. helophoroides*, the natural enemy of the trunk-boring pests.

## Conclusion

Overall, our experiment highlights that locomotor activity and oviposition capacity are influenced by the LD cycle and temperature and that chronic exposure to dim artificial light at night decreased the oviposition capacity of the beetle *D. helophoroides*. These findings enhance our understanding of the behavioral responses of *D. helophoroides* to different microhabitat environment. These finding also establish optimal microhabitat conditions for mass rearing of *D. helophoroides*, thus prolonging the shelf life or increasing the number of eggs laid.

## Data Availability

The raw data supporting the conclusion of this article will be made available by the authors, without undue reservation.
