# Supplementary material for: Exposure to artificial light at night mediates the locomotion activity and oviposition capacity of Dastarcus helophoroides (Fairmaire)
Source: Front Physiol. 2023 Feb 9;14:1063601. doi: 10.3389/fphys.2023.1063601 (PMC9947650; doi:10.3389/fphys.2023.1063601)
Supplement: Supplementary file 1 [file Table1.DOCX]

**Exposure to artificial light at night mediates the locomotion activity and oviposition capacity of *Dastarcus helophoroides* (Fairmaire)**

Xiang-lan Jiang^1^†, Zhe Ren^1^†, Xiao-xia Hai^1^, Ling Zhang^2^, Zhi-gang Wang^1^, Fei Lyu^1^^[[1]](#footnote-1)^*

1 Key Laboratories for Germplasm Resources of Forest Trees and Forest Protection of Hebei Province, College of Forestry, Agricultural University of Hebei, Baoding, Hebei 071000, P. R. China

2 Chengde Academy of Agriculture and Forestry Sciences, Chengde, Hebei 071000, P. R. China

**Supporting information**

**Appendix S1.** Statistical tests of locomotor activity percentages of adults between light phase and dark phase for different conditions.

.

Table S1 Student’s t test of differences in the locomotor active percentages of adults between the light phase and dark phase under different LD cycles and constant light and darkness.

| LD cycles | Light phase | Dark phase | *t* | *p* |
| --- | --- | --- | --- | --- |
| 0L/24D | - | 24.24 ± 7.62 | - | - |
| 4L/20D | 25.81 ± 9.11 | 38.53 ± 3.71 | 4.089 | 0.001 |
| 8L/16D | 16.28 ± 6.17 | 38.80 ± 7.51 | 7.321 | < 0.001 |
| 12L/12D | 24.06 ± 5.36 | 38.19 ± 2.35 | 7.627 | < 0.001 |
| 16L/8D | 31.64 ± 5.04 | 76.94 ± 4.40 | 21.411 | < 0.001 |
| 20L/4D | 37.50 ± 6.31 | 80.06 ± 4.28 | 17.642 | < 0.001 |
| 24L/0D | 12.50 ± 4.47 | - |  |  |

Table S2 Student’s t test of difference in the locomotor active percentages of adults between the light phase and dark phase at different temperatures.

| Temperatures (℃) | Light phase | Dark phase | *t* | *p* |
| --- | --- | --- | --- | --- |
| 15 | 11.95 ± 4.03 | 49.88 ± 4.11 | 20.832 | < 0.001 |
| 20 | 13.86 ± 2.72 | 57.72 ± 4.40 | 26.809 | 0.001 |
| 25 | 32.42 ± 4.93 | 75.38 ± 4.96 | 19.425 | < 0.001 |
| 30 | 36.39 ± 5.61 | 79.56 ± 3.69 | 20.357 | < 0.001 |
| 35 | 25.00 ± 11.90 | 44.19 ± 11.57 | 3.507 | 0.003 |
| 40 | 4.59 ± 3.12 | 4.84 ± 2.62 | 0.194 | 0.848 |

1. * Correspondence to: F. Lyu, College of Forestry, Agricultural University of Hebei, BaoDing, Hebei 071000, China. E-mail: haimolv[@foxmail.com](mailto:@foxmail.com).

   †: X.L Jiang and Z. Ren contributed equally to this work. [↑](#footnote-ref-1)
